# Tumor Hypoxia as a Barrier in Cancer Therapy: Why Levels Matter

**DOI:** 10.3390/cancers13030499

**Published:** 2021-01-28

**Authors:** Tord Hompland, Christina Sæten Fjeldbo, Heidi Lyng

**Affiliations:** 1 Department of Radiation Biology, Norwegian Radium Hospital, Oslo University Hospital, 0424 Oslo, Norway; Tord.Hompland@rr-research.no (T.H.); Christina.Saten.Fjeldbo@rr-research.no (C.S.F.); 2 Department of Physics, University of Oslo, 0371 Oslo, Norway

**Keywords:** hypoxia level, tumor microenvironment, model system, imaging, oxygen sensing, cellular response, radiotherapy resistance

## Abstract

**Simple Summary:**

Hypoxia is a common feature of solid tumors and associated with poor outcome in most cancer types and treatment modalities, including radiotherapy, chemotherapy, surgery and, most likely, immunotherapy. Emerging strategies, such as proton therapy and combination therapies with radiation and hypoxia targeted drugs, provide new opportunities to overcome the hypoxia barrier and improve therapeutic outcome. Hypoxia is heterogeneously distributed both between and within tumors and shows large variations across patients not only in prevalence, but importantly, also in level. To best exploit the emerging strategies, a better understanding of how individual hypoxia levels from mild to severe affect tumor biology is vital. Here, we discuss our current knowledge on this topic and how we should proceed to gain more insight into the field.

**Abstract:**

Hypoxia arises in tumor regions with insufficient oxygen supply and is a major barrier in cancer treatment. The distribution of hypoxia levels is highly heterogeneous, ranging from mild, almost non-hypoxic, to severe and anoxic levels. The individual hypoxia levels induce a variety of biological responses that impair the treatment effect. A stronger focus on hypoxia levels rather than the absence or presence of hypoxia in our investigations will help development of improved strategies to treat patients with hypoxic tumors. Current knowledge on how hypoxia levels are sensed by cancer cells and mediate cellular responses that promote treatment resistance is comprehensive. Recently, it has become evident that hypoxia also has an important, more unexplored role in the interaction between cancer cells, stroma and immune cells, influencing the composition and structure of the tumor microenvironment. Establishment of how such processes depend on the hypoxia level requires more advanced tumor models and methodology. In this review, we describe promising model systems and tools for investigations of hypoxia levels in tumors. We further present current knowledge and emerging research on cellular responses to individual levels, and discuss their impact in novel therapeutic approaches to overcome the hypoxia barrier.

## 1. Introduction 

**Figure 1:**
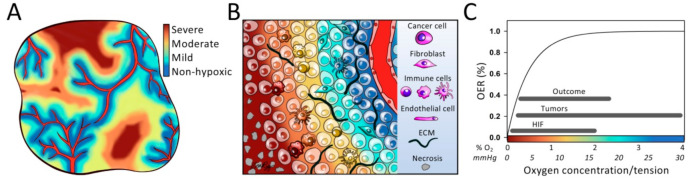
Distribution of hypoxia levels in tumors. (**A**) Illustration showing distribution of hypoxia levels from mild to severe in a section through a solid tumor. Non-hypoxic levels are seen close to capillaries (in red). (**B**) Close-up of a region in (**A**), showing the gradient in hypoxia levels from a capillary towards severe hypoxia. Symbols for different cell types and extracellular matrix (ECM) are indicated. (**C**) Illustration of how oxygen enhancement ratio (OER) increases with increasing oxygen concentration or tension in tumors, where OER is defined as the ratio between the dose needed to cause the same harmful effect to cells under anoxia and when oxygen is present. Data relative to maximum OER are shown and the curve is based on the study by Koch and coworkers described in [3]. The ranges indicated refer to the scale of the *x*-axis and are median pO_2_ values reported across tumor types, median pO_2_ cutoff for hypoxic fractions associated with poor radiotherapy outcome across tumor types, and median oxygen concentration reported for activation of hypoxia inducible factors (HIF). The pO_2_ data are collected from [5] and the HIF data are based on [6,7]. (**A**–**C**) Hypoxia levels are indicated by the color code, with approximate oxygen concentrations (% O_2_) and tensions (mmHg) provided by the *x*-axis in (**C**).

Solid tumors generally show regions with insufficient oxygen supply, defining them as hypoxic [1]. The oxygen distribution is highly heterogeneous with hypoxia levels ranging from mild, almost non-hypoxic, to severe and anoxic levels. This heterogeneity shows transient and long-term changes as the cancer develops [2], creating a dynamic pattern of hypoxia levels that induces cellular responses and controls interactions between tumor cells, stroma and immune cells in the microenvironment (Figure 1A,B). Hypoxia is an adverse factor associated with poor outcome in most cancer types and treatment modalities. The significance of various hypoxia levels for cancer treatment is well demonstrated by the higher cell kill of sparsely ionizing radiation in the presence of oxygen compared to under anoxic conditions, where a steep decrease in radiosensitivity is seen when the level changes from mild hypoxia of about 2% O_2_ to severe hypoxia of below 0.02% O_2_ (Figure 1C) [3]. In addition, individual hypoxia levels induce biological responses, like cancer cell survival and metastasis, that impair the treatment effect [4], and there is a large difference in the hypoxia level most strongly associated with poor radiotherapy outcome within and across cancer types (Figure 1C) [5]. This knowledge documents the importance of incorporating hypoxia levels in the work to understand treatment resistance mechanisms and implement new, upcoming therapeutic approaches, like immunotherapy, and radiation therapy with particles or in combination with hypoxia targeting drugs.

Our understanding of the heterogeneity in oxygen concentration in solid tumors originates from the report by Thomlinson and Gray in 1955 [8], describing an oxygen gradient from necrosis to capillaries in a human lung carcinoma. Later, by using polarographic needle electrodes to assess oxygen tension (pO_2_), Gatenby and coworkers [9] showed a relationship between pO_2_ in head and neck lymph node metastases and response to radiotherapy. This work provided the first clinical evidence for an adverse effect of hypoxia on treatment outcome. They defined a pO_2_ cutoff of 8 mmHg (about 1% O_2_) to calculate fraction of hypoxic tumor tissue, thereby introducing a level which defined absence or presence of hypoxia. Succeeding pO_2_ studies have demonstrated considerable differences within and across cancer types in the median hypoxia level, ranging from 2 mmHg to almost non-hypoxic levels of 32 mmHg, or approximately 0.2% to 4.2% O_2_ (Figure 1C) [5], and there is no universal cutoff to define hypoxia in tumors. Although this endorsement, hypoxia is now almost exclusively considered as a binary metric. The research field has moved away from direct pO_2_ measurements by needle electrodes to indirect measures by imaging, immunohistochemistry, and gene expression profiling, and hypoxia is defined from an arbitrary threshold or in analysis against treatment outcome [10]. 

Work in cell cultures has provided fundamental insight into how various hypoxia levels are sensed by cells and control their behavior. The importance of this knowledge was recognized by the award of the 2019 Nobel Prize in Physiology or Medicine for research on the hypoxia inducible factors (HIFs), which are major regulators of cell survival and activated already at mild hypoxia (Figure 1C) [11]. In recent years, an increased awareness of the role played by hypoxia in the interaction between cancer cells, stroma and immune cells has emerged [12]. It is further acknowledged that this role can be crucial in treatment resistance [13,14]. Quantification of hypoxia levels in tumor models and patients is necessary to fully understand these interactions and to design new treatments that fit the distribution of levels in individual patients. Here, we present an overview of model systems and methods that have shown promise for this purpose. We further review current knowledge of biological responses in cancer cells and the tumor microenvironment to individual hypoxia levels, and discuss possible implications of hypoxia levels for the success of new therapeutic approaches. 

## 2. Finding the Appropriate Model System

**Figure 2:**
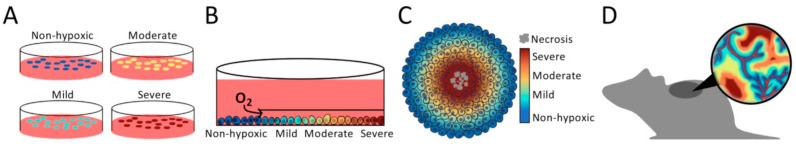
Model systems for studying hypoxia levels. (**A**) 2-dimensional (2D) monolayer cell cultures. Exposed to specific oxygen concentrations in a gas chamber. (**B**) 2D monolayer cell culture with an oxygen gradient created by a plate inserted into the petri dish at one end of the culture to limit oxygen and nutrient exchange with media. (**C**) Spheroid with a gradient in hypoxia levels from the periphery (non-hypoxic) towards the center (necrosis). (**D**) Animal model, showing a mouse with tumor grown on the back. The distribution of hypoxia levels in a section through the tumor is indicated. (**A**–**D**) hypoxia levels are indicated by the color code, with approximate oxygen concentrations (% O_2_) and tensions (mmHg) provided by the *x*-axis in Figure 1C.

The most common and yet powerful model is 2-dimensional (2D) monolayer cell cultures exposed to specific oxygen concentrations in a gas chamber (Figure 2A) [15,16]. This experimental setup facilitates measurement of biological responses in cells, cell lysates or culture medium at defined hypoxia levels. Moreover, co-cultures of cancer cells with fibroblasts or immune cells are feasible to model interactions between cell types, and can also be grown in 3D gel-based assays [17]. Cell line studies have been invaluable in our understanding of hypoxia responses, and important findings, such as the hypoxia level needed for stabilization of the HIF1 α-subunit (HIF1A), were first identified in this model system [7]. 

Standard 2D cell cultures fail to reproduce the oxygen gradient from a blood vessel in the tumor microenvironment. Such gradients have been modeled by culturing cells in a small chamber that connects to a larger volume of fresh media through a slit [18]. This limits oxygen and nutrient exchange with media on one end of the culture and creates gradients due to cell consumption and waste product secretion. The spatial position of cells determines oxygen availability (Figure 2B). This and similar systems have been used to investigate the spatial organization of a co-culture with cancer cells and macrophages and how the cells migrate in oxygen and nutrient gradients [18,19]. 

In spheroids and organoids, the microenvironment is more similar to in vivo conditions, including 3D cell-cell interactions and oxygen gradients from the surface towards the center (Figure 2C) [20]. Both models can incorporate co-cultures of different cell types, but organoids show a more complex and relevant tissue architecture than spheroids [21]. By use of immunohistochemistry and the hypoxia marker pimonidazole, biological responses like cell proliferation and DNA damage have been investigated at different hypoxia levels, e.g., [22]. To increase the reproducibility in 3D cell culture models, engineered 3D printed scaffolds have been presented, and used for studying cell metabolism at different hypoxia levels [23]. New models to mimic in vivo conditions in cell culture systems are rapidly emerging and can in many cases replace animal models, creating an experimental setup that enables careful control of hypoxia levels [24]. Still, animal models, such as xenografts and tumors grown in genetically engineered and syngeneic rodents, are indispensable in hypoxia studies due to the importance of host tissue interactions [25]. 

## 3. Quantification of Hypoxia Levels

### 3.1. Invasive Methods 

**Figure 3:**
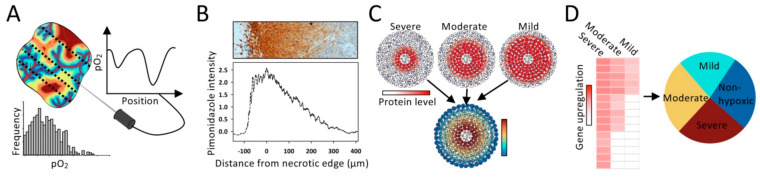
Invasive methods for quantification of hypoxia levels. (**A**) Polarographic needle electrodes for recording of pO_2_ along tracks in a tumor. Frequency distribution of recorded pO_2_ values is indicated. (**B**) Pimonidazole staining intensity in a histologic section from a xenograft tumor vs. distance from necrosis (below). The histologic section is shown above. (From “MRI Distinguishes Tumor Hypoxia Levels of Different Prognostic and Biological Significance in Cervical Cancer”. by Hillestad, T.; Hompland, T.; Fjeldbo, C.S.; Skingen, V.E.; Salberg, U.B.; Aarnes, E.-K.; Nilsen, A.; Lund, K.V.; Evensen, T.S.; Kristensen, G.B.; et al. 2020, Cancer Res., 80, 3993–4003, Copyright 2020 by American Association for Cancer Research [27]). (**C**) Spheroids indicating proteins upregulated at different hypoxia levels (above), and the combined expression data (below). (**D**) Gene expression signatures associated with defined hypoxia levels (left), and pie chart showing fractions of tumor with the defined level (right). (**A**,**C**,**D**) Hypoxia levels are indicated by the color code, with approximate oxygen concentrations (% O_2_) and tensions (mmHg) provided by the *x*-axis in Figure 1C.

Polarographic needle electrodes provide a direct recording of pO_2_, and have served as the gold standard for measuring hypoxia in tumors (Figure 3A) [5]. In the 1980s, the Eppendorf pO_2_ histograph was launched, and pO_2_ distributions could be assessed even in patient tumors, by moving the electrode in steps of around 0.7mm along several tracks in the tissue [26]. The electrodes are not feasible for routine use and not commonly applied today. A large number of clinical studies has, however, been accomplished, providing a valuable documentation of the range of hypoxia levels in human tumors and normal tissues, and how large the hypoxia problem is in the clinic for most cancer types and treatment modalities like radiotherapy, chemoradiotherapy and surgery [5]. 

Nitroimidazole compounds like pimonidazole and pentafluropropyl (EF5) are powerful tools for assessment of hypoxia levels in tumors (Figure 3B). Nitroimidazoles are chemically reduced in hypoxic cells, forming adducts that bind irreversibly to macromolecules and can be detected by immunohistochemistry or immunofluorescence [28]. The binding increases exponentially with decreasing oxygen concentration [29] below about 1.3% O_2_ (10 mmHg) [30,31], although staining has been detected at higher concentrations up to about 9.2% O_2_ (70 mmHg) [32]. A wide range of hypoxia levels can therefore be assessed, and a steady decrease in staining intensity away from necrosis in histological tumor sections has been shown (Figure 3B) [27,33]. By comparing this staining pattern with expression data of proteins like epidermal growth factor receptor (EGFR) and HIFA, molecular responses at different hypoxia levels have been assessed [34,35]. Such studies have further shown that individual hypoxia-responsive proteins are not suitable as endogenous markers of hypoxia [36], possibly because their expression depends on temporal fluctuations in oxygen concentration and the presence of other metabolic stressors like lactate, acidosis and glucose deprivation [2,37,38]. However, a promising approach combining expression data of multiple proteins upregulated at different hypoxia levels has been presented and may be more robust than individual proteins (Figure 3C) [39]. By combining co-registered microcopy images of osteopontin (OPN), glucose transporter member 1 (GLUT1), and HIF1A protein expression, an image reflecting pimonidazole staining intensity and, hence, hypoxia levels was obtained. This technique can be applied on paraffin embedded archive material without prior administration of nitroimidazole compound. The expression level of multiple hypoxia-responsive genes has further been combined into cancer type specific signatures that have shown great promise as biomarkers of treatment outcome [40]. Most signatures are based on gene responses to a single oxygen concentration in cell cultures [40] or the binary hypoxia status of tumors, as assessed by pO_2_ measurements [41], pimonidazole staining intensity [42] or medical imaging [43], with no focus on hypoxia levels. The hypoxia level(s) represented by the signatures are therefore not known. It may be possible, however, to construct signatures that predict a defined hypoxia level by careful consideration of genes or proteins responsive only to the selected oxygen concentration (Figure 3D). 

### 3.2. Non-Invasive Imaging for Preclinical Studies

Imaging is an appealing technology, since information about the entire tumor is achieved and changes in hypoxia levels over time or in response to treatment can be recorded. The requirement for spatial resolution depends on the heterogeneity of the levels and has not been studied in detail. For accurate measurement of the absence or presence of hypoxia, a resolution below one mm has been shown to be needed [44]. With suboptimal resolution, several hypoxia levels are averaged in each voxel and misleading results are achieved.

**Figure 4:**
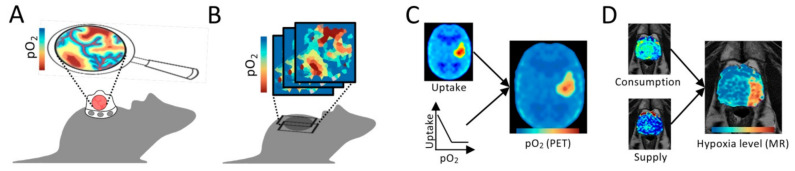
Imaging of hypoxia levels. (**A**) Optical pO_2_ imaging of tumor in a dorsal skinfold chamber. The image with hypoxia level distribution is indicated. (**B**) Electron paramagnetic resonance (EPR) imaging of a tumor grown on the mouse back. Images with hypoxia level distribution are indicated. (**C**) Illustration of a positron emission tomography (PET) image showing uptake of hypoxia specific tracer (left), and pO_2_ image based on the converted PET signal (right). The relationship between uptake (PET signal) and pO_2_ is indicated. (**D**) Diffusion weighted (DW) magnetic resonance (MR) images showing apparent diffusion coefficient (ADC), reflecting oxygen consumption, (left, upper) and fractional blood volume (fBV), reflecting oxygen supply (left, lower), and the combined hypoxia level image (right). The images were collected from a patient with prostate cancer, and the DW-MR images are overlaid on T_2_ weighted images of the pelvis. (**A**–**D**) Hypoxia levels are indicated by the color code, with approximate oxygen concentrations (% O_2_) and tensions (mmHg) provided by the *x*-axis in Figure 1C.

In a preclinical setting, optical pO_2_ imaging based on oxygen induced quenching of phosphorescence, is an exciting approach (Figure 4A) [45]. By recording phosphorescence lifetimes after excitation of porphyrin probes by light, a pO_2_ image averaged for all depths, with a high accuracy and spatial resolution of 2.4 μm in superficial tumors, is acquired [46]. Due to the poor penetration depth of about 0.7 mm of light in tissues [47], studies have been mostly limited to tumors in window chambers. Despite this limitation, new knowledge of how for example pH [48] and T-cell motility [46] relate to oxygen gradients in tumors has been obtained. Recently, a technique combining phosphorescence quenching with excitation by Cherenkov light was presented and shown feasible for imaging of tumors down to six mm of depth [47]. The Cherenkov light was generated after high-energy radiation during fractionated radiotherapy. Furthermore, near infrared excitable nanoprobes have been developed for pO_2_ imaging of depths up to one cm [49,50]. 

Electron paramagnetic resonance (EPR) can be used for 3D imaging of pO_2_ in tissue depths of up to 5–10 mm; however, with the cost of a lower spatial resolution down to about one mm (Figure 4B). The technique is based on the relaxivity of oxygen, using a paramagnetic spin probe as contrast agent. By combining the approach with MR metabolic imaging based on hyperpolarized ^13^C-labeled pyruvate, the oxygen dependent antitumor effect of the glycolysis inhibitor 3-bromopyruvate was examined [51]. New knowledge of how different hypoxia levels affect cellular responses to a glycolysis inhibitor was obtained, demonstrating a potential of this technique in preclinical interventional studies.

### 3.3. Medical Imaging 

There are no established methods for medical imaging of hypoxia levels in patient tumors; however, promising experimental positron emission tomography (PET) and magnetic resonance (MR) based techniques are currently applied in clinical trials to assess hypoxia status. Although PET suffers from poor spatial resolution, with a typical voxel size of 64 mm^3^ for clinical scanners and 8 mm^3^ for preclinical micro-PET scanners [52], an important advantage of this technique is the existence of several hypoxia specific tracers. Most of them are based on nitroimidazole compounds and demonstrate similar oxygen dependent binding kinetics as pimonidazole [53]. The tracer uptake is therefore directly related to hypoxia level (Figure 4C) [54,55,56]. Frequently used nitroimidazole tracers, ^18^F-fluoromisonidazole (^18^F-FMISO) and ^18^F-fluoroazomycin-arabinoside (^18^F-FAZA), show half maximum binding at about 0.1–0.3% O_2_ (0.8-2.1 mmHg) in vitro [57,58,59]. This relationship between oxygen concentration and tracer uptake has been used to convert PET signal to pO_2_ [54], although without validation against other measures of hypoxia levels. The tracer binds intracellularly, and normalization of the signal with paired data on cell density obtained from a biopsy or MR image would be a useful approach to assess hypoxia levels. 

MR images have high spatial resolution and superior soft tissue contrast, with opportunities for signal weighting to enhance specific tissue properties. Oxygen concentration cannot be measured directly, but parameters related to hypoxia can be imaged. For extracting hypoxia levels, tissue oxygen-level-dependent (TOLD) MR imaging (MRI), which utilizes that the presence of dissolved oxygen causes a decrease in the longitudinal relaxation time (T_1_), has been proposed. By using a T_1_ weighted sequence, signal enhancement after inhalation of 100% O_2_ can be detected and is interpreted as an increase in tissue oxygenation [60]. It is hypothesized that voxels with enhanced signal represent non-hypoxic tissue at baseline while voxels with no enhancement represent hypoxic or necrotic tissue; however, this remains to be proven. It should also be clarified whether the signal enhancement in response to oxygen inhalation, i.e., about 1–5% out of baseline signal [60], is large enough to distinguish specific hypoxia levels.

Images reflecting oxygen consumption and oxygen supply, like cell density and blood volume or perfusion, respectively, can be constructed by quantitative analysis of diffusion weighted (DW) and dynamic contrast enhanced (DCE) MR images [61]. We have developed a tool termed consumption and supply based hypoxia (CSH) imaging to combine two such images voxel-by-voxel into a single hypoxia image by machine learning (Figure 4D) [62]. The success of combining multiparametric MR images to assess hypoxia has later also been demonstrated by others [63]. Recently, we used the CSH tool to construct an image visualizing a continuous distribution of hypoxia levels from mild to severe in cervical cancer [27]. CSH imaging thus enables imaging of hypoxia levels at a high resolution with a typical voxel size of 3 mm^3^ for clinical scanners and 0.042 mm^3^ for preclinical scanners, by exploiting MR equipment already available in the clinic. In combination with measures of cellular responses in biopsies [27] or PET images with molecular tracers, this approach appears to be a promising tool to investigate the biology underlying different hypoxia levels in patient tumors.

## 4. Biological Significance of Hypoxia Levels

**Figure 5:**
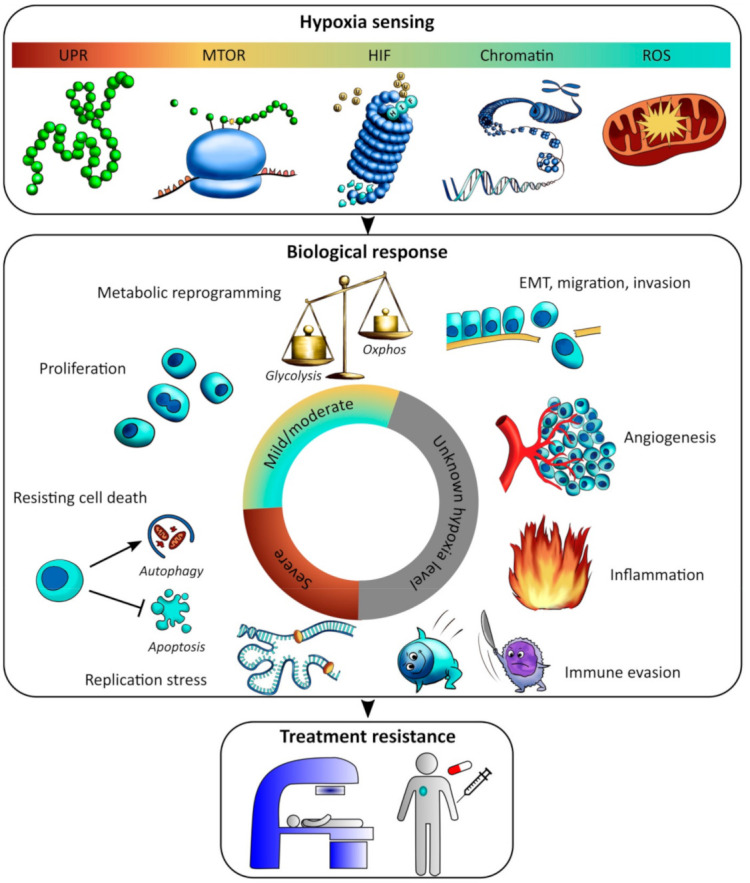
Hypoxia sensing and response. Illustration of hypoxia sensing mechanisms (upper), biological responses (middle) in relation to hypoxia level from mild to severe, and subsequent treatment resistance (lower). HIF, Hypoxia-inducible factor; UPR, unfolded protein response; MTOR, mammalian target of rapamycin; ROS, reactive oxygen species; EMT, epithelial-mesenchymal transition. Hypoxia levels are indicated by the color code, with approximate oxygen concentrations (% O_2_) and tensions (mmHg) provided by the *x*-axis in Figure 1C. Unknown hypoxia level is indicated in grey.

Cell function is maintained through transcriptional and translational activities, which are highly energy-consuming processes [64]. Under hypoxia, tumor cells reprogram their activity from general housekeeping functions to activation of specific pathways, aiming to conserve energy under the deprived conditions. The biological responses are initiated by several hypoxia sensing mechanisms, and determine whether the cells will survive and thus become a treatment resistant subpopulation of the tumor, or die (Figure 5).

Most of our understanding in this field comes from in vitro studies where cells are grown as 2D monolayer cultures, which may limit their validity for tumors in vivo in some cases as described in Section 2 and Section 3. Graded responses with decreasing oxygen concentration without any clear cutoff are generally reported, and the responses often depend on hypoxia exposure time and show variations across cell lines. Moreover, cell lines have adjusted to 21% O_2_ over time in culture and the choice of non-hypoxic control is not straightforward [65]. In addition, overlap may exist between hypoxia sensing mechanisms and other stress-induced pathways, which allows non-hypoxic stresses to activate hypoxia-inducible responses. Despite these precautions, it is possible to define some sensing mechanisms and responses specific for mild and moderate hypoxia and others for more severe levels (Figure 5). 

### 4.1. Hypoxia Sensing at Mild and Moderate Levels

At mild and moderate hypoxia in the range between 3% and approximately 0.5% O_2_, the overall protein synthesis is slowed down, whereas transcription of selected genes is escalated. The best understood sensing mechanism at this level controls the HIF family of transcription factors. The HIFs are major regulators of gene expression under hypoxia with numerous target genes involved in processes like metabolism, angiogenesis, pH regulation and proliferation [66]. The stability and transcriptional activity of the α-subunit of HIFs are negatively regulated by hydroxylation by dioxygenase enzymes, which depend on oxygen for activity [67]. Under non-hypoxic conditions, this subunit is hydroxylated and targeted for degradation by the von Hippel–Lindau (VHL) protein. Under hypoxia, hydroxylation is reduced, the α-subunit is stabilized, and HIF is activated [67]. Stabilization of the HIF1 and HIF2 subunits HIF1A and EPAS1 is seen at oxygen concentrations as high as 2% O_2_ [6,7]. HIFs are also regulated by oxygen independent pathways [68]. In tumor sections only a weak correlation between HIF1A expression and oxygen level by pimonidazole staining or electrode measurements has been found, e.g., [35,69,70,71], and expression of this protein alone is not a robust indicator of hypoxia level, as discussed in Section 3. In particular, in kidney cancer, HIFs are generally constantly active, also in non-hypoxic tumor regions, due to VHL loss of function [72].

Mild hypoxia induces changes in chromatin conformation that also affect transcriptional activity, although the effect on downstream signaling is not completely understood [73]. Chromatin acts as an accessibility barrier for transcription; a closed conformation with nucleosomes tightly packed blocks the accessibility of transcriptional regulators and silences transcription, while a more open conformation allows transcription. Histones are a core part of the chromatin, and different histone methylations are associated with open or closed chromatin. Specific members of the lysine demethylase (KDM) family of epigenetic regulators are dioxygenases and inhibited at low oxygen concentrations, coordinating transcriptional changes under hypoxia. In macrophages, increased histone methylation has been observed below 3% O_2_ and was associated with transcriptional downregulation of chemokines [74]. Moreover, in two complementary studies, this sensing mechanism was shown to be HIF independent and proceed subsequent transcriptional events [75,76].

Release of reactive oxygen species (ROS) by the mitochondria at oxygen concentrations in the mild hypoxia range further regulates transcriptional responses [77,78], apparently in an HIF-independent manner [79]. A more than doubling in ROS production has been reported when the oxygen concentration is lowered from 21% to 2% O_2_ in cancer cells [80,81] and a four-fold increase is seen at 0.5% O_2_ [80]. ROS production stabilizes HIF1A in cancer cells [82] and various normal tissue cells [83,84]. Different mechanisms seem to be involved [85,86,87,88], and stabilization by ROS under non-hypoxic conditions has also been demonstrated, e.g., [89]. Other molecular responses have been linked to hypoxia-induced ROS production as well, including upregulation of the AMP activated protein kinase (AMPK) [90], which promotes glycolysis.

Overall protein synthesis is slowed down to conserve energy even before oxygen becomes metabolically limiting. At moderate hypoxia of 1.5% O_2_, cap-dependent mRNA translation is repressed independent of HIF, by inhibition of mammalian target of rapamycin (MTOR) signaling [91,92]. Cells thus need alternative pathways to efficiently synthesize proteins from newly transcribed hypoxia-responsive genes, but these mechanisms are still under debate [93]; for example, selective localization of specific mRNAs, including HIF1 targets, to the endoplasmatic reticulum (ER) or selective alteration of translation efficiency has been demonstrated at 1% O_2_ [94,95]. In epithelial cells exposed to 1% O_2_, it has further been shown that EPAS1 remains in the cytoplasm to initiate selective cap-dependent translation of hypoxia-responsive proteins [96], suggesting that HIF plays a role in this process.

### 4.2. Boosting of Selected Activities at Mild and Moderate Hypoxia

Oxygen concentration is still sufficient to retain strictly selected activities at mild and moderate hypoxia. For energy production, anaerobic glycolysis is favored over oxidative phosphorylation, although oxygen is not a limiting factor for oxidative phosphorylation until around 0.4–0.7% O_2_ [97,98,99]. This metabolic reprogramming is controlled mainly by the transcriptional activity of HIFs. Elevated glucose uptake in cells is seen at reduced oxygen concentration in the mild hypoxia range [100,101]. Moreover, increased gene or protein expression of glucose transporter GLUT1 and key enzymes in the glycolytic pathway, including HK2, GAPDH, ALDOA and ENO1 has been reported at 1.5–1% O_2_, e.g., [100,102,103,104]. Moreover, enzymes that deviate pyruvate from the tricarboxylic acid (TCA) cycle by excessive lactate formation are upregulated at these oxygen concentrations, including lactate dehydrogenase A (LDHA), which converts pyruvate to lactate, and the monocarboxylate transporter MCT4, which export lactate out of cells [102,105]. Many of these findings have been confirmed in tumor sections in vivo, showing increased GLUT1, LDHA and MCT4 protein expression at increasing distance from vessels and strong expression in pimonidazole positive areas and around necrosis [106,107]. Mild and moderate hypoxia also induces other metabolic changes, including increased glutaminolysis to sustain fatty acid synthesis, as mediated by HIF1-dependent gene expression changes [108]. 

The elevated glycolytic activity also seems to play a role in apoptosis resistance, and thus facilitates cell proliferation under hypoxic conditions [109,110,111]. While severe hypoxia (<0.1% O_2_) induces growth arrest, moderate hypoxia (1% O_2_) allows for continued or even increased proliferation [111,112,113] in a cell line dependent manner [114]. Moreover, co-localization of proliferating cells and pimonidazole staining has been demonstrated in tumor sections [42,115], most likely representing cells in moderate hypoxic regions. 

### 4.3. Overall Shut Down and Activation of Survival Strategies at Severe Hypoxia 

Severe hypoxia, below about 0.5% O_2_, constitutes a huge danger for tumor cells, and energy-consuming processes including translation are more strongly suppressed to protect against lethal effects [116]. Further, disulfide bonds introduced during post-translational folding of proteins are oxygen-dependent and inhibited at concentrations lower than 0.3% O_2_ [117]. Unfolded or misfolded proteins thus accumulate in the ER lumen and lead to activation of ER stress sensors in the unfolded protein response (UPR). This inhibits global protein synthesis, but also induces selective translation of mRNAs required to restore ER homeostasis and sustain survival, promoting hypoxia tolerance [116,117,118,119,120,121]. Total inhibition of global protein synthesis occurs at levels close to anoxia, while modest inhibition is seen at 0.2% O_2_ [116,120], consistent with the oxygen sensitivity of disulfide bond formation [117]. 

MTOR suppression and UPR activation slow down or stop many cellular activities. DNA synthesis is retarded due to stalling of replication forks, leading to severe replication stress [122]. Nuclear expression of phosphorylated histone H2A variant H2AX (ƴH2AX) is indicative of replication stress under hypoxia and is strongly induced below 0.1% O_2_ [123], helping to recruit repair factors and protect cells from DNA damage [124]. Arrest in S-phase of the cell cycle and accumulation of single stranded DNA in the nuclei have been demonstrated at concentrations below 0.02% O_2_ [125], and these levels also arrest cells in late G_1_-phase [126]. The mechanisms behind stalling of replication forks are not completely understood, but a depletion of ribonucleotide pools in hypoxic cells seems to be important [122]. The oxygen-requiring ribonucleotide reductase (RNR) enzyme provides cells with deoxyribonucleotides (dNTPs) for S-phase replication. This enzyme responds to limited oxygen concentrations below 0.1% O_2_ by switching a subunit of the enzyme to a version that helps maintaining sufficient dNTP for ongoing replication [127]. However, due to low activity of this enzyme, the generated dNTPs are insufficient for normal replication rate [122]. 

Severe hypoxia (<0.1% O_2_) further induces a replication-associated DNA damage response in the absence of DNA damage, by activation of the damage response transducer gene ataxia-telangiectasia mutated (ATM). Bencokova and coworkers [123] showed ATM activation at 0.02% O_2_, but not at 0.5% O_2_. Activation was independent on HIF1A [123]; however, chromatin modification may be involved [128]. In addition, DNA repair pathways, including homologous recombination (HR) and non-homologous end-joining, are downregulated at severe hypoxia [129], possibly due to lack of repair proteins [123]. Hence, prolonged (48 h) exposure of cells to 0.01% O_2_ induced a 6-fold decrease in expression of the HR repair protein RAD51 that was apparent also at 0.5% O_2_ and accompanied by a decrease in HR [130]. 

If tumor cells become reoxygenated after being exposed to extreme hypoxia, replication restart combined with impaired repair capacity can lead to extensive DNA damage and genomic instability [131]. Thus, increased mutation rate and breaks at fragile DNA sites have been detected in cells exposed to anoxia (<0.001% O_2_), followed by reoxygenation [132,133,134]. Moreover, these growth conditions have been shown to increase the metastatic potential of cells when injected into the vein of mice [135,136,137], whereas exposure to 0.2% O_2_ or higher had no such effect [136]. Further, the effect on metastasis was associated with a high degree of DNA overreplication after reoxygenation [137]. Downregulation of DNA repair pathways under severe hypoxia (<0.2% O_2_) can also hamper repair of G_1_-associated DNA double strand breaks in irradiated cells, resulting in increased genomic instability after radiation [138].

Suppression of apoptosis and promotion of autophagy are key cell survival strategies at severe hypoxia [118,139,140]. Hypoxia can induce apoptosis at oxygen concentrations below about 0.5% O_2_, depending on the expression of apoptosis inducers like tumor protein 53 (TP53) and members of the BCL2 apoptosis regulator family [112,140,141]. Hypoxia thus serves as a selection pressure, expanding subpopulations with diminished apoptotic potential [140]. In addition, autophagy facilitates survival through recycling of cellular components for maintaining ATP production and macromolecular synthesis. Autophagy is induced over a wide oxygen concentration range from moderate (1% O_2_) to extreme hypoxia (<0.02% O_2_) [118,139,142], and is linked both to HIF1 and UPR signaling [118]. Autophagic cells co-localize with hypoxic tumor regions in histological sections [118,139,143] and can be observed throughout the full pimonidazole gradient without being restricted to perinecrotic regions, i.e., the most severe hypoxia levels [118]. Moreover, mitochondrial autophagy reduces oxygen consumption and can be induced at moderate hypoxia (1% O_2_) in a HIF1-dependent manner, preventing ROS production and subsequent cell death [144]. Below this level, there is a steep decrease in the cellular respiration rate [97,98,99]. 

## 5. Involvement of the Tumor Microenvironment

Hypoxia-responsive processes like cell migration and invasion, angiogenesis, inflammation, and immune evasion, involve a complex interplay between cancer cells, stroma and immune cells (Figure 5). Thus, in addition to the impact of hypoxia on cancer cells, its influence on the surrounding cells and extracellular matrix is important. This is a relatively new field of research, and the critical oxygen concentrations are largely unknown.

### 5.1. Epithelial-Mesenchymal Transition (EMT), Migration and Invasion

Hypoxia drives invasion and migration of tumor cells through processes such as EMT and upregulation of genes involved in extracellular matrix modulation [145,146,147,148,149,150,151,152], possibly in a HIF1A-dependent manner [145]. The hypoxia level investigated varies and includes 0.5% [145,146], 1% [147,148,150,151,152], and 2% O_2_ [149]. In a 3D model where small sarcoma grafts or cells were encapsulated in oxygen-controlled hydrogels, mimicking the gradients seen in vivo, a hypoxic gradient (0.5–4% O_2_) promoted invasion and migration towards increased oxygen concentrations compared to a non-hypoxic gradient (10–15% O_2_) [153]. Studies comparing the effect of different hypoxia levels are, however, rare. 

### 5.2. Angiogenesis

Tumor angiogenesis is a coordinated process in response to hypoxia that involves cancer cells and endothelial cells, as well as fibroblasts and immune cells [154]. Proangiogenic factors secreted by the cells stimulate vessel growth. Although aiming to restore oxygen homeostasis, tumor vasculature is chaotic and poorly organized, without leading to relief of hypoxia. Most oxygen sensing mechanisms seem to be involved [155], but the importance of individual hypoxia levels is not well known. Human umbilical vein endothelial cells (HUVEC) show a steady increase in expression of the pro-angiogenic vascular endothelial growth factor A (VEGFA) from moderate hypoxia of 1% O_2_ to severe hypoxia of 0.1% O_2_ [156]. Severe hypoxia (0.2% O_2_) also induces VEGF expression in macrophages [157], whereas half maximum expression in cancer cell lines has been found to occur at mild and moderate levels from 2.7–1.3% (27–13 µM O_2_) [158]. 3D co-cultures of HUVEC and cancer associated fibroblasts (CAFs) in a gel-based assay demonstrated that hypoxia (1% O_2_) promotes angiogenesis, and that secreted factors from hypoxia-treated CAFs, including VEGFA, are involved in this effect [17].

### 5.3. Inflammation and Immune Evasion

The tumor immune response is a balance between antitumor mechanisms and the protumor inflammatory response. Hypoxia is an important inducer of the inflammatory response, which contributes to immune evasion and cancer progression by recruiting protumor immune cells and inhibiting antitumor immune cells [159,160]. These effects are mediated through both HIF1A-dependent and independent mechanisms [161]. Infiltration of immunosuppressive cells, including tumor-associated macrophages (TAMs), myeloid-derived suppressor cells (MDSCs) and regulatory T-cells (Treg) has been demonstrated in hypoxic tumor regions, and evidences point to a role of hypoxia in their immunosuppressive functions [162,163,164,165,166]. Macrophages can develop into a protumor phenotype by exposure to severe hypoxia (0.2% O_2_) [157]. Such cells have been found to accumulate in perinecrotic regions [167] and promote inhibition of T-cells at 1% O_2_ in a HIF1A-dependent manner [168]. 

Cytotoxic (CD8+) T-cells are important mediators of the antitumor immune response. Although the reported direct effects of hypoxia on T-cells vary, a reduced antitumor effect is generally seen [160], and hypoxic tumor regions are poorly infiltrated by T-cells [169,170]. Respiratory hyperoxia in mice breathing 60% oxygen has been shown to promote spontaneous tumor regression and reduce number of metastasis, and this effect was attributed to increased infiltration of T-cells in regions with reduced hypoxia after the intervention [169]. T-cells have further been found to be less motile below about 0.7% O_2_ (5 mmHg), possibly reducing their efficacy of cancer cell killing in hypoxic tumor areas [46]. Moreover, hypoxia at 0.5% and 1% O_2_ inhibits T-cell proliferation and effector function through reduced generation of interferon type 2, IFN-γ [171,172,173]. However, the suppressive effect of hypoxia on the T-cell antitumor response seems to be largely driven by changes on tumor cells [160]. These changes include downregulation of major histocompatibility complex class I (MHC I) molecules [171], inhibition of INF-γ-stimulated gene expression [171], and induced expression of the immune inhibitory molecule programmed cell death ligand-1 (PD-L1) [174], which have been demonstrated at 0.5% O_2_. Moreover, 1% O_2_ has been shown to decrease T-cell mediated tumor cell lysis through induction of autophagy in the tumor cells [143].

## 6. Perspectives

### 6.1. Advancing Biological Understanding of Hypoxia Levels 

Detailed understanding of hypoxia sensing and responses of cancer cells grown at different oxygen concentrations has been obtained, but our knowledge of how hypoxia levels influence the cellular interactions within the tumor microenvironment is scarce (Figure 5). Molecular crosstalk between different cell types makes the oxygen-dependency profiles more difficult to establish for such processes, and advanced tumor models like cells grown in co-cultures and 3D models are required (Figure 2). A challenge in this research is that hypoxia often co-localizes with metabolic stressors like lactate, acidic pH and/or glucose deprivation in the microenvironment and shows transient fluctuations, which can influence the hypoxia-related cellular responses [2,37,38]. These effects should be integrated into the investigations of hypoxia levels. 

#### 6.1.1. Multiparametric and Multimodality Imaging

An exciting opportunity lies in the co-registering of hypoxia level images (Figure 4) with images of biological responses. The potential of this approach was demonstrated in the work by Rytelewski and coworkers [46], using intravital microscopy to combine optical images of hypoxia levels with fluorescence images of T-cell motility in a window chamber tumor model. This approach allowed studies of T-cells motility in relation to oxygen concentration and provided evidence for impaired motility at severe hypoxia. Furthermore, by subjecting animals to 100% oxygen gas breathing, they observed a rapid alleviation of hypoxia and increase in T-cell motility. Hence, such combination of images provides a high-resolution tool for preclinical studies of static and dynamic responses in relation to oxygen gradients. Moreover, hypoxia levels can be manipulated in order to verify causal relationships. 

**Figure 6:**
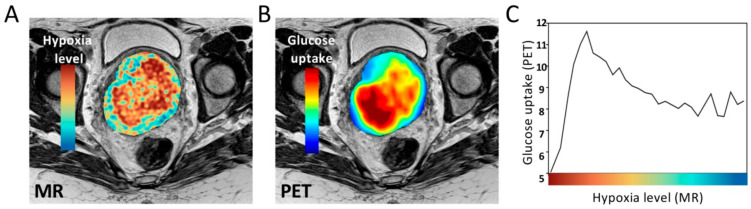
Glucose uptake at different hypoxia levels by combination of multimodality images. (**A**) Magnetic resonance (MR)-based hypoxia level image of a cervix tumor. (**B**) Glucose uptake in the same tumor by ^18^F-fluorodeoxyglucose (^18^F-FDG)-positron emission tomography (PET) imaging. (**C**) Glucose uptake as a function of hypoxia level based on a voxel-by-voxel analysis of the co-registered images in (**A**,**B**). The hypoxia levels were divided into 20 sublevels, and the mean PET signal of each sublevel is plotted. (**A**,**B**) The hypoxia level and glucose uptake images are overlaid on T_2_ weighted images of the pelvis. (**A**–**C**) Hypoxia levels are indicated by the color code, with approximate oxygen concentrations (% O_2_) and tensions (mmHg) provided by the *x*-axis in Figure 1C.

For assessment of hypoxia levels in deeper lying tumors or tumors in patients, medical imaging is particularly attractive (Figure 4). Most patients undergo comprehensive multiparametric MR and PET imaging as a part of their diagnostic procedure. These techniques can therefore rapidly be used in large scale studies without high costs or changes in the hospital’s infrastructure. Such methods will likely increase our understanding of hypoxia levels within and across patient tumors in the future. In addition, co-registering of images makes it possible to combine images of hypoxia levels on a voxel-by-voxel basis with medical images showing other biological tumor features. An example is seen in Figure 6, where an MR-based hypoxia level image of a cervix tumor, derived as described in [27], was combined with a ^18^F-fluorodeoxyglucose (^18^F-FDG)-PET image of glucose uptake. In this tumor, glucose uptake increased from mild towards more severe hypoxia, consistent with studies in cell lines [100,101], and was followed by a steep decrease at the most severe levels. The approach can be extended by using upcoming PET tracers to visualize immune responses [175] or drug uptake [176]. 

#### 6.1.2. Molecular Characterization of Tumor Samples

Large scale investigations of molecular features associated with different hypoxia levels can be performed by combining imaging with genome wide characterization of biopsies from the same tumors, in a similar manner as for images reflecting the absence or presence of hypoxia [177]. Such studies are possible based on patient material, utilizing images and biopsies collected routinely at diagnosis. In recent work, we combined MR-based hypoxia level images with gene expression profiles in cervical cancer patients and identified a set of genes for which expression correlated with individual hypoxia levels [27]. Further exploration of the correlating genes in cancer hallmark analysis revealed distinct biological processes associated with each hypoxia level, including proliferation at moderate hypoxia and EMT, inflammation and angiogenesis at the most severe levels. The potential of this approach was also demonstrated by combining imaging and HIF1A immunohistochemistry, showing the strongest correlation at mild hypoxia [27]. The data for mild and moderate hypoxia are consistent with current knowledge (Figure 5), while the results for severe hypoxia provide hypotheses for further investigations in experimental studies.

A similar strategy is to utilize hypoxia level images by immunohistochemistry of nitroimidazole compounds (Figure 3B) in combination with genome wide characterization of abutting tissue sections. This would be a feasible extension of studies where the absence or presence of hypoxia, and not the levels, has been considered, including our work to identify a gene expression signature associated with pimonidazole staining in prostate cancer [42].

### 6.2. New Treatment Options

#### 6.2.1. Radiation Delivery Techniques

Increasing the radiation dose to tumor as a strategy to overcome hypoxia-related radioresistance, is in most cases not feasible due to normal tissue dose constraints. However, a redistribution of the dose by escalating the dose to the radioresistant tumor regions only, has been proposed. Such dose-painting techniques are currently being evaluated in clinical trials where a higher dose is generally prescribed to hypoxic regions defined in PET images by a cutoff value [178]. However, hypoxia level images together with the radiation response curve in Figure 1C enable construction of radioresistance images that most likely would be more appropriate. In addition, combined devices of MR imaging and linear accelerators (MR-linacs) are on the way into clinical routine [179]. MR-based methods to image hypoxia levels during radiation treatment might facilitate dose-painting with these machines, and development of such methods are likely to be pursued in the coming years. Moreover, it is possible that persistent hypoxia or lack of reoxygenation during the course of fractionated radiotherapy is more important for outcome than the pretreatment hypoxia level [180,181]. Hypoxia level images would be valuable for testing this hypothesis and determine whether a change in hypoxia levels during the early phase of radiotherapy could be a stratification factor for dose escalation studies.

Particle therapy based on protons, and in some cases carbons, are currently being established worldwide [182]. Protons offer more precise radiation delivery than photons, and therefore an exciting opportunity for dose-painting based on high resolution hypoxia level imaging. Moreover, recent research indicates that protons produce more complex DNA damage than photons at the end of the Bragg peak where the energy deposition is denser, and that cells possibly need different DNA repair mechanism to survive these damages [182]. A focus on hypoxia levels in the work to reveal these repair mechanisms would be important [182] and may help to understand the biological effects of protons in patient tumors.

Ultrafast delivery of the radiation dose, termed FLASH radiotherapy, is a new and intriguing delivery technique where the dose rate is several orders of magnitude higher than with conventional radiation [183]. This ultrafast delivery has been shown to reduce normal tissue toxicity compared to conventional radiation, while tumor responses remain the same. The mechanisms behind these observations are not understood; however, FLASH irradiation may deplete oxygen and induce hypoxia during parts of the delivery period [183,184], thereby increasing radioresistance. Since the tumor is inherently more hypoxic than normal tissues, the sparing effect would be higher in the surrounding normal tissue. However, tumors exhibit a wide range of hypoxia levels, including levels outside the range of maximum radioresistance (Figure 1C), and some tumors will likely be spared similar to normal tissue. If the above hypothesis is true, hypoxia level imaging would be of utmost importance for identifying patients who will benefit from FLASH irradiation.

#### 6.2.2. Combination Therapies with Hypoxia Targeting Drugs

Hypoxia is an attractive drug target in combination with radiotherapy and chemoradiotherapy [185]. Promising candidates are nitroimidazole compounds, hypoxia-activated prodrugs, and drugs aiming to increase tumor oxygenation by targeting oxidative phosphorylation. Clinical studies have shown limited success, except for the combination of nimorazole and chemoradiotherapy in head and neck cancer [186]. Nimorazole works by fixating and stabilizing the radiation induced DNA damage in hypoxic tumor regions in the same way as oxygen under non-hypoxic conditions. The drug shows the highest radiosensitizing effect in cell lines at severe hypoxia or anoxia, while the effect is minor at moderate levels of about 1.5% O_2_ [187]. The pro-drugs are activated in cells by an enzymatic reduction reaction, which is inhibited by oxygen. Like nimorazole, the pro-drugs evofosfamide (TH-302) and PR104A have the highest activity at severe hypoxia (0.1% O_2_), while for tirapazamine, this is achieved at moderate levels of 0.6–1.5% O_2_ [188,189]. Moreover, drugs targeting oxidative phosphorylation [190], like the anti-diabetic agent metformin, will probably be most effective at mild and moderate levels where mitochondrial activity is still significant (Figure 5). Thus, there are differences in the optimal oxygen concentration across drugs, and therefore likely in their effect on hypoxia-related radioresistance. This emphasizes the need to report such data for new candidates. Moreover, the distribution of hypoxia levels should be recorded to enable selection of the drug that fits the distribution of each patient.

#### 6.2.3. Immunotherapy and Combination Therapy with Radiation

Immunotherapies have emerged as an important treatment modality in cancer care. However, many patients do not respond, and evidence points to hypoxia as a player also in the resistance to this therapy [170,172,191], most likely because hypoxia suppresses the antitumor immune response. Thus, reducing tumor hypoxia using hypoxia-activated prodrugs [170], or metformin [172] have shown increased benefit of immune checkpoint blockade in preclinical models. For understanding the role of hypoxia in the resistance to immunotherapy and for optimizing the treatment effect, knowledge of the hypoxia levels involved would be crucial.

Tumor immune evasion is associated with poor radiotherapy outcome [14], and the combination of radiation with immunotherapy is a novel, promising strategy to overcome radioresistance [192]. Radiotherapy can itself induce antitumor immune responses; however, the combination with immunotherapy is believed to boost such responses and enhance the radiation effect. The role of hypoxia in this combination treatment has hardly been addressed. Partial radiation of bulky tumors by targeting exclusively hypoxic regions with stereotactic body radiation (SBRT) has shown both local and distant, non-targeted effects [193], which have been attributed to activation of antitumor immune responses [194]. It is most likely that the response to radiation and immune checkpoint blockage combined will depend on the hypoxia level, and that this promising strategy can be further optimized by using hypoxia level images for prescription of the radiation dose.

## 7. Conclusions

A large body of knowledge exists on how various hypoxia levels affect tumor biology. Several hypoxia sensing mechanisms have been identified, but their individual link to the cellular responses is not completely understood. For example, it is not clear how a mechanism like chromatin modification, being activated already at mild hypoxia, can mediate cellular responses, such as DNA damage signaling, at severe hypoxia. In addition, more efforts are needed to reveal how individual hypoxia levels control interactions between cancer cells and the microenvironment. Powerful model systems and technologies are available for this purpose, including upcoming medical imaging approaches for investigations in patients. Such investigations can reveal which levels that are most important for therapy outcome [27]. This is important to better understand the therapeutic window of each treatment modality and develop more robust biomarkers for patient selection. A stronger focus on the distribution of hypoxia levels—rather than the absence or presence of hypoxia—in our investigations will further help in designing new therapeutic approaches to overcome the obstacles associated with each level. In this way, we will more likely see successful clinical trials aiming to overcome the hypoxia barrier in cancer treatment.

## Data Availability

Data sharing is not applicable to this article.
